# Esophagectomy for esophageal stricture with systemic sclerosis: a case report

**DOI:** 10.1186/s40792-023-01727-3

**Published:** 2023-11-10

**Authors:** Yuho Ebata, Yasue Kimura, Kentaro Nonaka, Sho Nambara, Qingjiang Hu, Ryota Nakanishi, Tomonori Nakanoko, Mitsuhiko Ota, Eiji Oki, Tomoharu Yoshizumi

**Affiliations:** Department of Surgery and Science, Graduate School of Medical Sciences, Kyushu University, 3-1-1, Maidashi, Higashi-Ku, Fukuoka, 812-8582 Japan

**Keywords:** Systemic sclerosis, Gastroesophageal reflux disease, Esophageal stricture, Esophagectomy

## Abstract

**Background:**

Systemic sclerosis (SSc) is an autoimmune disease characterized by frequent esophageal involvement. However, there are few reports on esophagectomy for esophageal strictures associated with SSc. Herein, we present a case of successful treatment of an esophageal stricture associated with SSc through subtotal esophagectomy.

**Case presentation:**

A 53-year-old female patient was diagnosed with SSc, interstitial pneumonia, and gastroesophageal reflux disease (GERD). The patient developed an esophageal ulcer and benign stricture that required a subtotal esophagectomy 10 years after the diagnosis. Histopathological findings revealed thinning of the muscle layer, a characteristic feature of SSc. The patient was free of dysphagia or regurgitation.

**Conclusions:**

An esophagectomy is a valuable option for treating esophageal strictures in SSc. Therefore, surgical approaches should be established for patients with SSc.

**Supplementary Information:**

The online version contains supplementary material available at 10.1186/s40792-023-01727-3.

## Background

Systemic sclerosis (SSc) is a complex autoimmune disease in which the small arteries become fibrotic, and collagen is deposited in multiple organs [1]. There are two major types of SSc: the limited and diffuse types. The latter can affect a wide area of the skin and one or more internal organs [1]. Most patients with SSc often have esophageal involvement (90%) and gastroesophageal reflux disease (GERD) [2, 3]. There have been some reports of esophagectomies for esophageal strictures in patients with SSc; however, definitive indications for surgical treatment are lacking [4]. We report a case of esophageal stricture formation in SSc that was successfully treated with an esophagectomy and present a review of the current literature on esophagectomies for esophageal strictures in patients with SSc.

## Case presentation

**Fig. 1:**
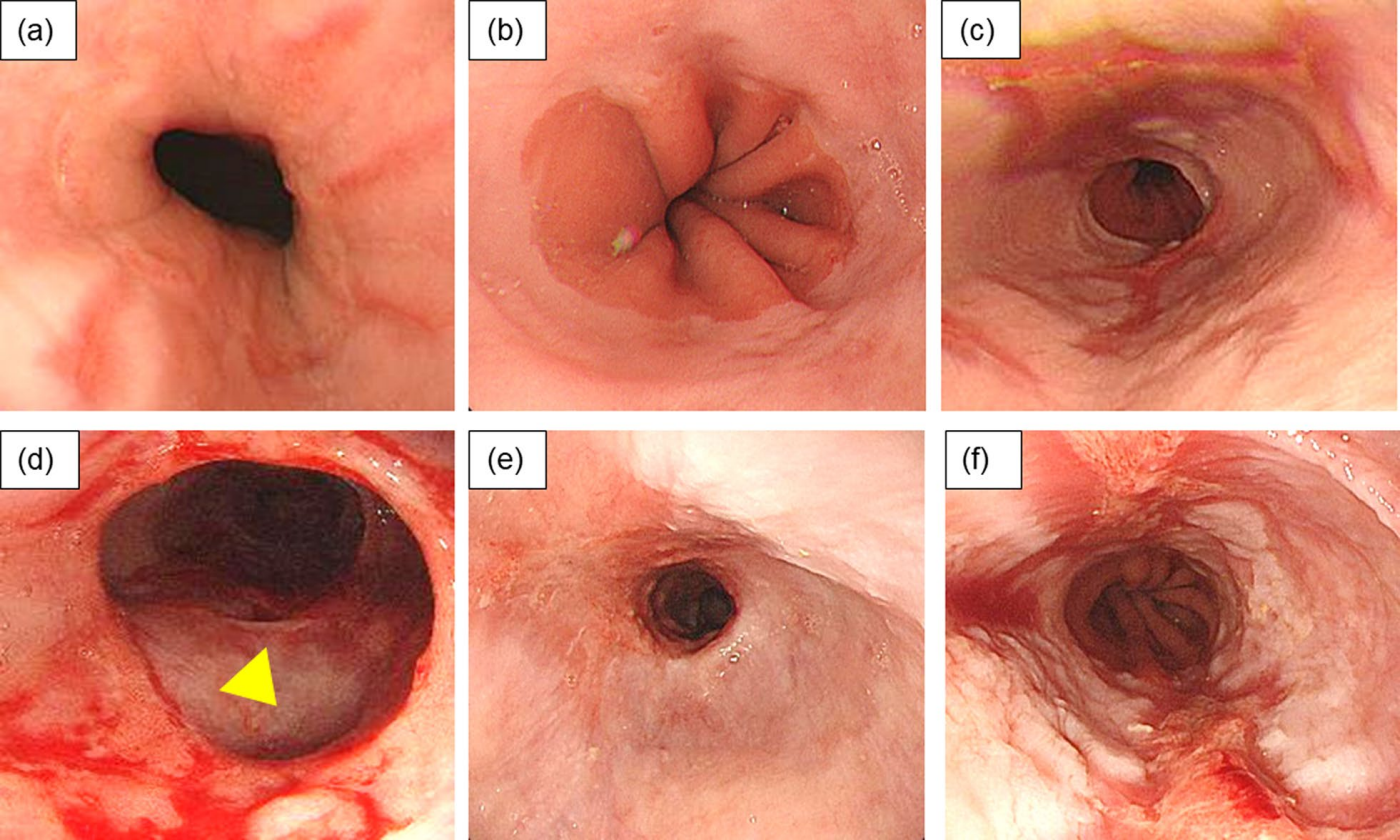
Endoscopy showed a reflux esophagitis of Los Angeles classification Grade B 15 years ago (**a**). Endoscopy revealed Grade M reflux esophagitis 5 years ago (**b**), but it further worsened to Grade C 3 years ago (**c**). Endoscopy showed 20 mm esophageal ulcer (arrowhead) and benign stenosis due to scar tissue in the middle thoracic esophagus 1 year ago (**d**). Endoscopy revealed restenosis 30 cm from the incisors despite multiple balloon expansions and Grade D reflux esophagitis when the patient was referred to our department (**e**, **f**)

**Fig. 2:**
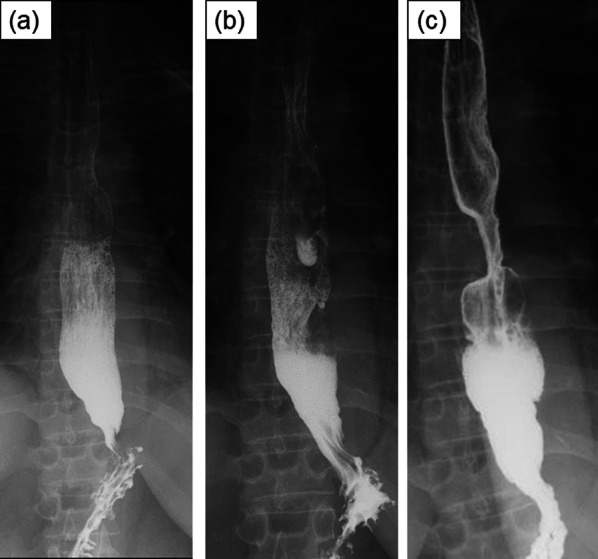
Esophagogram showed no expansion or constriction but the esophagus was in a state of aperistalsis 5 years ago (**a**), but it remained in a state of aperistalsis 3 years ago (**b**). Esophagogram revealed an esophageal stricture 30 mm in the middle thoracic esophagus (**c**)

A 53-year-old female patient with SSc was referred to our department for further evaluation and treatment of an esophageal stricture. The patient was diagnosed with SSc with associated GERD (Fig. 1a) and interstitial pneumonia (IP) and had been taking medication continuously for the past 15 years, including a proton pump inhibitor (PPI), mycophenolate mofetil (1.5 g), tacrolimus (3 mg), and prednisolone (5 mg). The patient had been undergoing regular checkups but developed symptoms of reflux and cough and was admitted for examination 5 years ago. Endoscopy revealed mild reflux esophagitis and an esophageal hiatus hernia (Fig. 1b). Esophageal manometry showed that the integrated relaxation pressure (IRP) was 14.3 mmHg and a complete absence of peristalsis. Esophagography showed no peristalsis; however, there was an obstruction (Fig. 2a). The 24-h multichannel intraluminal impedance-pH (24 h MII-pH) monitoring revealed significant non-acid reflux. A small bowel contrast study indicated weak peristalsis of the small intestine. The patient's medication was adjusted, and her condition remained stable.

However, the patient’s dysphagia worsened, and esophagography revealed esophageal aperistalsis and liquid passage disturbance, leading to readmission for examination 3 years ago (Fig. 2b). Endoscopy revealed moderate reflux esophagitis (Fig. 1c), and no stricture or dilation was detected. Esophageal manometry confirmed the aperistalsis; the lower esophageal sphincter pressure (LESP) was 14.2 mmHg, and the IRP was 6.8 mmHg, which was lower than in the previous examination. The PPI dose was increased, and the patient was discharged.

The GERD symptoms had worsened a year ago despite ongoing medication. Endoscopy revealed severe reflux esophagitis and ulceration (Fig. 1d). The IRP and LESP were 5.8 and 12.6 mmHg, respectively, on manometry. The 24-h MII-pH monitoring could not provide an accurate evaluation, because the esophagus was filled with liquid. The patient had developed a membranous stricture and edema of the middle thoracic esophagus 3 months after the previous examination. A radical incision and cutting (RIC) procedure was performed to alleviate the severe dysphagia without any adverse events.

After treatment, the patient remained stable for 6 months; however, stricture recurrence occurred, and another RIC procedure was performed. As the progression of stenosis worsened despite treatment, the patient underwent several endoscopic balloon dilations; however, the procedures were unsuccessful. Finally, the patient was referred to our department for surgery.

On admission, the patient had severe dysphagia, regurgitation, skin induration, and tightening of the face and fingers. Endoscopy revealed severe reflux esophagitis and esophageal stenosis (Fig. 1e, f), and esophagography confirmed the presence of a stricture (Fig. 2c). Contrast-enhanced computed tomography (CT) revealed dilatation and liquid pooling in the proximal esophagus and a stable fibrosing non-specific interstitial pneumonia pattern in the lungs (Additional file 1: Fig. S1a, b). There were no atypical cells on biopsy. With good control of the IP, the cardiopulmonary function was normal.

**Fig. 3:**
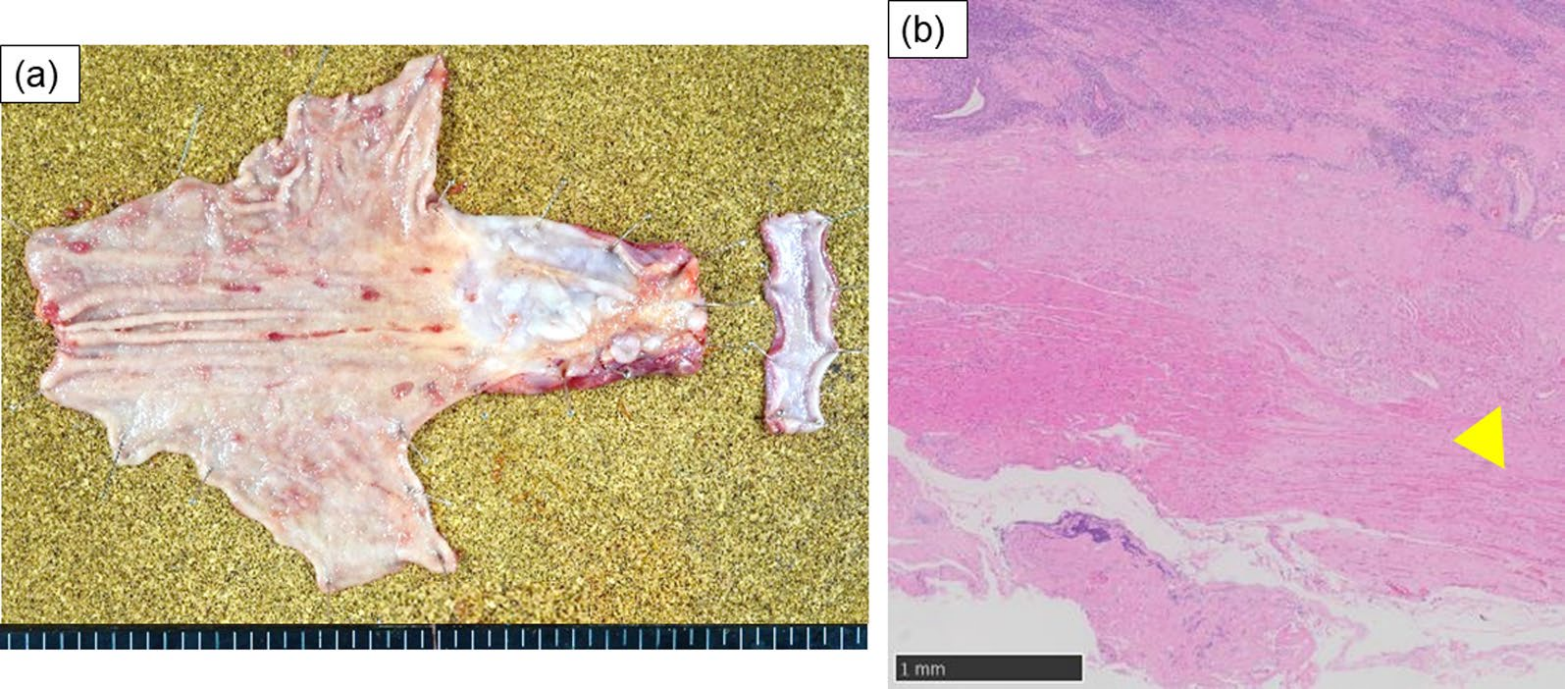
Macroscopic finding of esophageal stenosis, 5.0 × 3.0 cm in size (**a**). Microscopic examination revealed thinning of the muscular layer and an increase in fibrous tissue (**b**, (arrowhead). (Hematoxylin and eosin stain, 10 ×)

Endoscopic treatments for the severe esophageal stricture caused by GERD and SSc, were unsuccessful. Therefore, the patient underwent thoracoscopic subtotal esophagectomy and laparoscopic gastric tube reconstruction through the retrosternal route. Despite multiple endoscopic treatments, no adhesions were observed around the esophagus. The operation time was 318 min, and the blood loss was 80 mL without any adverse events. The surgical specimen contained an esophageal stricture 50 × 30 mm in size (Fig. 3a), and histopathological examination showed thinning and tearing of the muscular layer and fibrous tissue replacement of the muscle tissue (Fig. 3b).

After the surgery, pneumothorax occurred due to inadequate air drainage, but it was successfully managed by inserting a chest tube for 3 days. In addition, the patient required a 1-month hospitalization for tacrolimus adjustment. Finally, the patient was discharged in stable condition. The patient continued regular checkups and showed no symptoms or findings of GERD or esophageal stricture 6 months after surgery.

## Discussion

Patients with SSc who have esophageal involvement often present with symptoms, such as dysphagia, heartburn, or reflux, which increase their risk of developing esophageal adenocarcinoma and reflux-related lung diseases [5]. According to the Japanese SSc guidelines, the severity of upper gastrointestinal tract lesions is classified into five stages from 0 to 4 [6]. Stage 0 is normal, and stage 4 indicates the most severe condition with dysphagia related to esophageal stenoses [6]. Esophageal involvement in SSc is known to occur in the middle to lower esophagus because of decreased peristalsis and lower LESPs caused by atrophy and fibrosis of the esophageal smooth muscle [7].^.^ [8] Most patients with SSc often have esophageal involvement; however, reports on esophagectomies are rare. The patients with SSc who underwent esophagectomies in our case and in seven previous reports [9–15] are demonstrated in Table 1. Sixteen cases have been reported, with a median age of 51 years and patients’ ages ranging from 18 to 63 years at the time of surgery. The patient consisted of 8 females (80%), and all patients had dysphagia. All stenoses were located in the middle to lower thoracic esophagus, except in cases where the location was not specified. In our case, the patient with SSc had a stable condition for 10 years but developed mild GERD 5 years ago, which worsened, causing an ulcer and esophageal stricture. An aperistaltic esophagus with fibrosis and atrophy was seen on manometry, along with a hiatal hernia, making reflux easier. Decreases in the IRP and LESP worsen GERD, leading to refractory GERD, ulcers, and strictures.

**Table 1:** Characteristics for esophageal stenosis with systematic sclerosis

Author, year	Age (year), sex	Number of patients	Symptom	Location	Stricture, length (cm)	Methods of esophagectomy	Reconstruction organ, route	Perioperative complication	Prognosis (months)
Our case, 2023	53, F	1	Dysphagia, regurgitation	Mt	( +), 5	Thoraco-laparoscopic surgery	Gastric tube, retrosternal	Pneumothorax	Alive (6)
Yekeler et al. [7], 2008	20, M	1	Dysphagia	Mt	( +), NR	Thoraco-laparoscopic surgery	Gastric tube, NR	None	Alive (24)
Kent et al. [8], 2007	NR	5	Dysphagia, heart burn	NR	( +) in 3 patients, NR	Invasive surgery (3 patients), open surgery (2 patients)	NR	Heart failure (1 patient), pneumonia (2 patients), anastomotic leak (2 patients), chylothorax (1 patient)	Alive in 4 patients (14*)
Kamal et al. [9], 1988	NR	1	Dysphagia	NR	( +), NR	NR	Colon, NR	None	Alive (NR)
Okuyama et al. [10], 1980	63, F	1	Dysphagia	Mt-Lt	( +), NR	Laparotomy without thoracotomy	Gastric tube, retrosternal	None	Alive (12)
Akiyama et al. [11], 1973	18, M	1	Dysphagia	Mt-Lt	( +), 7	Laparotomy without thoracotomy	Gastric tube, posterior mediastinal	None	Alive (4)
Brain et al. [12], 1973	54, F	1	Dysphagia	Mt-Lt	( +), NR	NR	Jejunum, NR	None	Died (108) Cardiac failure
	61, F	1	Dysphagia	Mt-Lt	( +), NR	NR	Jejunum, NR	None	Alive (120)
	59, F	1	Dysphagia	Mt-Lt	( +), NR	NR	Jejunum, NR	None	Alive (72)
	48, F	1	Dysphagia	Mt-Lt	( +), NR	NR	Colon, NR	Intrapleural hematoma	Alive (12)
Mc Laughlin et al. [13], 1971	47, F	1	Dysphagia	Lt	( +), NR	open surgery	Gastric tube, NR	None	Alive (NR)
	43, F	1	Dysphagia	Lt	( +), NR	open surgery	Gastric tube, NR	None	Alive (NR)

*F* female, *M* male, *Mt* middle thoracic esophagus, *Lt* lower thoracic esophagus, *NR* not reported

*Median (range)

The standard treatment for GERD is acid suppression medication; however, resistance or side effects can occur in patients with SSc. Therefore, surgical intervention may play a significant role [16]. As surgical methods for GERD, fundoplications such as Nissen, Toupet, or Dor and Roux-en-Y reconstruction are known, and the latter was shown to be the best method in a previous study [16]. However, if the patient has already developed an esophageal stricture, endoscopic balloon dilations and RIC procedure should be selected first. The RIC procedure was developed in 2013, and there are no reports of esophageal stricture in patients with SSc except in our case. Favorable outcomes have been reported in cases resistant to endoscopic balloon dilation for anastomotic strictures after surgery [17]. If multiple endoscopic treatments are unsuccessful, esophagectomy is a good option [9, 10]. All patients underwent esophagectomies, and the technique and organs for reconstruction varied (Table 1).

In our case, the patient had reflux symptoms for a long time; therefore, multiple fundoplications were considered; however, conservative treatment was chosen because of the risk of passage obstruction or intestinal disorders due to weak peristalsis of the esophagus and jejunum. However, the patient developed severe esophageal stenosis despite attempts at endoscopic treatment; therefore, a subtotal esophagectomy was performed. As adhesions around the esophagus were expected, the thoracoscopic method was chosen. Reconstruction was performed using a gastric tube, and a triangular anastomosis was created through the retrosternal route without severe adverse events. As shown in Table 1, the incidence of complications was relatively low, and only one patient died of right heart failure due to pulmonary hypertension immediately after esophagectomy [8].

The prognosis of patients with SSc with esophageal strictures is classified as mild gastrointestinal tract involvement, and the outcome is reported to be better than compared to severe involvement, such as pseudo-obstruction, malabsorption, or hyperalimentation [5]. Only one patient died 9 years after surgery [12] due to heart failure, but no patient died after discharge. In our case, the patient was stable for 6 months after surgery without any symptoms of dysphagia or regurgitation. Therefore, esophagectomy for esophageal strictures in patients with SSc may be a valuable option.

## Conclusion

Herein, we report a case of esophageal stricture with severe GERD in a patient with SSc successfully treated with esophagectomy and gastric tube reconstruction using thoracoscopic surgery. Evidence regarding the surgical treatment of patients with SSc is lacking; however, surgical treatment can be a valuable option.

### Supplementary Information

**Additional file 1: Fig. S1.** CT showed ground-glass opacities and traction bronchiectasis on both posterior aspects of the lungs, consistent with a fibrosing non-specific interstitial pneumonia pattern (a and b).

## Data Availability

Not applicable.

## Abbreviations

24-h MII-pH: 24-H multichannel intraluminal impedance-pH
CT: Computed tomography
GERD: Gastroesophageal reflux disease
IP: Interstitial pneumonia
IRP: Integrated relaxation pressure
LESP: Lower esophageal sphincter pressure
PPI: Proton pump inhibitor
RIC: Radical incision and cutting
SSc: Systemic Sclerosis
