# Supplementary figures and images for: Esophagectomy for esophageal stricture with systemic sclerosis: a case report
Source: Surg Case Rep. 2023 Nov 10;9:195. doi: 10.1186/s40792-023-01727-3 (PMC10635916; doi:10.1186/s40792-023-01727-3)

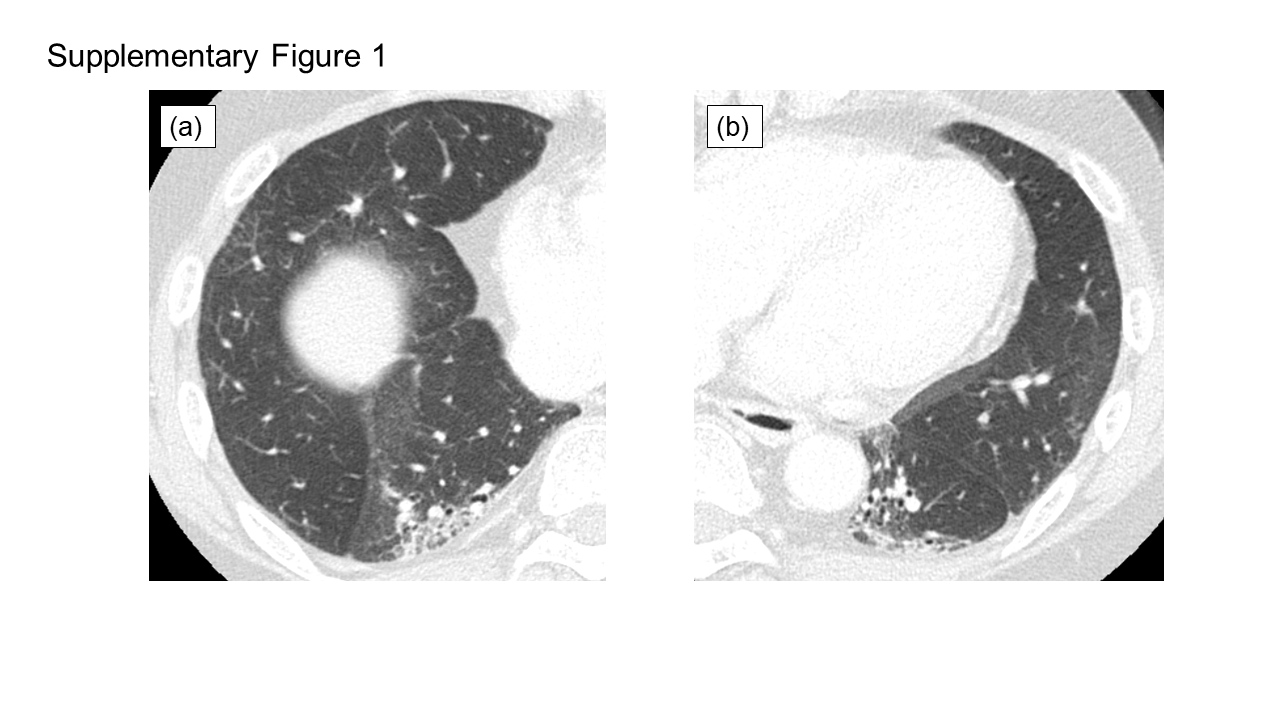

Supplement: Supplementary file 1 — Additional file 1: Fig. S1. CT showed ground-glass opacities and traction bronchiectasis on both posterior aspects of the lungs, consistent with a fibrosing non-specific interstitial pneumonia pattern (a and b). [file 40792_2023_1727_MOESM1_ESM.tif]
